# Is the Vertical Transmission of *Neotyphodium lolii* in Perennial Ryegrass the Only Possible Way to the Spread of Endophytes?

**DOI:** 10.1371/journal.pone.0117231

**Published:** 2015-02-13

**Authors:** Barbara Wiewióra, Grzegorz Żurek, Dariusz Pańka

**Affiliations:** 1 Department of Seed Science and Technology, Plant Breeding and Acclimatization Institute—National Research Institute, Radzików, Poland; 2 Departament of Grasses, Legumes and Energy Plants, Plant Breeding and Acclimatization Institute—National Research Institute, Radzików, Poland; 3 Department of Molecular Phytopathology, University of Technology and Life Sciences, Bydgoszcz, Poland; Fujian Agriculture and Forestry University, CHINA

## Abstract

Endophytic fungi live their whole life within host tissues usually without any visible symptoms. Their vertical transmission (seed-plant-seed) has been well described and documented. However, horizontal transmission (plant-plant) needs more clarification. The aim of this study was to assess the extent to which endophytes move vertically in ecotypes of perennial ryegrass and whether there is evidence for the horizontal transmission of endophytes. Ecotypes from grasslands in Poland were collected in the form of living plants and used for vertical transmission analysis. Plants, the seed collected from these plants and plants grown from this collected seed were tested for endophytic infection. Provided that all produced seeds were viable and able to germinate and produce seedlings, the vertical transmission of *Neotyphodium* endophytes in perennial ryegrass ecotypes was nearly complete. For the horizontal transmission experiment, endophyte-hosting plants (E+) and endophyte-free plants (E-) of four cultivars were planted in the field in close proximity on small plots that were frequently mown. These studies revealed that after 7 months of growth next to E+ plants, the characteristic *Neotyphodium* spp. mycelia were found in E- plants, which was especially true for plants growing in close proximity to the infected plants. The occurrence of horizontal transmission of endophytes has not been previously demonstrated.

## Introduction

Endophytic fungi subsist within host tissues usually without any visible symptoms [1]. The most widely known endophytes of the genus *Neotyphodium* are: *N. coenophialum* (Morgan-Jones and Gams) Glenn, Bacon and Hanlin, *N. lolii* (Latch, Christensen and Samuels) Glenn, Bacon and Hanlin and *N. uncinatum* (Gams, Petrini and Schmidt) Glenn, Bacon and Hanlin. These endophytes colonise tall fescue (*Schedonorus arundinaceus* (Schreb.) Dumort = *Festuca arundinacea* Schreb.), perennial ryegrass (*Lolium perenne* L.) and meadow fescue (*Schedonorus pratensis* (Huds.) P. Beauv. = *F. pratensis* Huds.), respectively. Although positive effects of fungus on host performance (e.g., increased pest, disease or drought resistance) are possible [2, 3, 4], documentation of the negative effect of endophyte-produced toxins on feeding animals is more common [5, 6, 7, 8, 9]. Most endophytes can reproduce sexually by producing ascospores [10, 11]. Asexual forms (anamorphs) of the genus *Neotyphodium* develop in the ripening seeds of the infected host [12], which allows this symbiosis to spread vertically into the next generation of plants. For estimations of the dynamics of natural grass population infection with respect to possible negative effects on animals, understanding the mechanisms of endophyte transmission is crucial.

Vertical transmission of endophytes has been well described and documented. It is claimed to be the only way of transmission for asexual *Neotyphodium* endophytes [13, 14]. In the case of seed production, significant differences in seed infection could be affected by the age of the crop used for seed production. In the first year of grass seed harvest, the infection of a seed population by *Neotyphodium* was shown to be less severe than the infection in the material used for establishing the plantation [15]. In the subsequent years of plantation use, increases in the number of seeds inhabited by endophytes were observed. In the following years of seed harvest, a similar increase of seed infection by an endophyte was reported by Cagaš [16]. In his studies, the upward trend of seed infection was clearly indicated when seeds with less severe infections were used for sowing. In the initial stages of infection, spreading does not always require full transmission of an endophyte, and therefore, the fungus is not present in all shoots produced by the plant [17]. Chlebicki [18] argues that endophytes might be lost during the transmission to the next generation because of endophyte instability and genetic isolation. In addition, many species of the genus *Neotyphodium* can act antagonistically with one another [19]. Hume *et al*. [20] reported that the sources of infection for new seed and fodder can also be endophyte-infected seeds from the soil seed bank, which might explain the observed annual growth of infection to some extent. Lewis [21] stated that a major factor affecting the presence of endophytes is seed plantation age, and older plantations display more severe plant infections by these fungi.

*Neotyphodium lolii*, when growing in plant, did not produce spores involved in horizontal transmission [22]. However, some authors suggest that strictly asexual *Neotyphodium* endophytes might be occasionally transmitted horizontally (i.e., plant-to-plant) because of the presence of epiphyllous nets and conidia in the leaves of some infected grasses [23]. It can be expected that if *Neoptyphodium* can be readily inoculated into E- plants in the laboratory with simple technique then infection may also be transmitted horizontally in nature [19]. However, this hypothesis has not been unequivocally demonstrated [19].

This study is of particular importance in Poland where the majority of grassland is still semi-natural, but endophytes are frequently found in plants and also in commercially available seed mixtures [24, 25, 26]. Grass species of the major importance in grasslands in Poland is perennial ryegrass. It is a primary grass used for pasture and silage in dairy and animal farms and as a soil stabilization plant. This species is the predominant forage and turf grass in Europe, and has been used in the United States, Japan, New Zealand and Australia for forage and lawns [27, 28]. Perennial ryegrass has several important characteristics which account for its widespread use and popularity. Among them are high herbage yield, a long growing season, tolerance to a wide range of environmental conditions and grazing, rapid establishment, persistence under close grazing and high forage quality an palatability [29].

The objectives of this study were to determine: (i) to what extent the vertical transmission of endophyte may occur in perennial ryegrass, and (ii) whether the *N. lolii* can be transmitted horizontally in perennial ryegrass.

## Materials and Methods

### Vertical transmission of *Neotyphodium lolii*

Studied ecotypes of perennial ryegrass were collected during 2007–2008 from permanent grasslands in the form of living plants. Approximately 5 to 10 individuals, naturally grown in distances of 5 to 10 meters were collected per one ecotype in each site. Three regions of Poland were sampled: Podlaskie (POD), Mazowieckie (MAZ) and Świętokrzyskie (SWK). No specific permissions were required for these locations. GPS coordinates of 18 location are presented in Table 1.

**Table 1:** Mean endophyte infection (%) of perennial ryegrass ecotypes originating from Podlaskie (POD), Świętokrzyskie (SWK) and Mazowieckie (MAZ) regions in Poland.

Region	Total number of ecotypes:		Average E+ frequency for region	Selected ecotypes:			GPS coordynates	
	collected	E + with endophytes		no of ecotype	Se [%]	Ee [%]	N	E
POD	16	9	56.2%	05/07	6.0	8.0	52°70.161	21°90.503
				50/07	62.0	57.4	53°65.353	23°13.897
				27/07	17.0	18.9	53°04.803	23°64.958
				03/07	15.0	11.1	52°70.161	21°90.503
				105/07	2.0	3.7	53°41.794	21°67.781
				101/07	46.0	38.9	53°41.872	21°67.719
average for region:					24.7	23.0	x	x
SWK	14	11	78.6%	45/08	100.0	94.4	50°84.056	21°92.464
				129/08	99.0	100.0	50°60.492	20°50.658
				87/08	100.0	90.6	50°42.564	20°55.994
				273/08	73.0	70.4	50°80.289	20°43.508
				227/08	61.0	64.2	50°53.908	20°93.531
				160/08	100.0	98.1	50°68.544	20°73.581
average for region:					88.8	86.3	x	x
MAZ	16	9	56.2%	685/08	83.0	70.4	52°04.600	21°30.189
				1350/08	9.0	9.6	52°02.964	22°59.114
				873/08	100.0	98.1	52°82.650	21°49.408
				131/08	89.0	87.0	52°19.578	22°48.228
				730/08	76.0	64.8	51°70.561	21°61.714
				801/08	75.0	61.1	52°37.494	20°37.808
average for region:					72.0	65.2	x	x
LSD (P>95%)					31.1	28.5

Explanation: Se – level of seeds infection in %, Ee – level of plants infection in %

The collected ecotypes were maintained at the Plant Breeding and Acclimatization Institute—National Research Institute in Radzików, Poland. After few months, well established and developed plants were screened for endophyte presence. Ten tillers from each plant were tested. Small epidermal strips were peeled off the adaxial surface of the leaf sheaths and placed on a glass slide into a drop of rose Bengal staining solution [30]. After 1 min, stained epidermal strips were covered with a cover-glass slip and examined microscopically at 100–400× magnification. The endophyte appeared as an intercellular, long and convoluted hyphae running parallel to the leaf-sheath axis of the plant cell without forming haustorial structures [31]. Results of the examination are presented as a percentage of the ecotypes of a given region, exhibiting symptoms of endophyte presence.

Six ecotypes of the highest level of plant infection were selected from each region for greenhouse experiment. Seeds collected from these ecotypes during the next growing season were analyzed for endophyte presence according to Saha *et al*. [30]. Seeds were soaked in a 5% NaOH solution for 8–16 h for softening. Then, the solution was removed by rinsing with tap water for 20 min and seeds were placed in a rose Bengal staining solution for about 3–4 h. Soft, stained seeds were then rinsed with distilled water, placed on a microscope slide, squashed with a cover slip and examined microscopically at 100–400× magnification. A seed was considered to be E+ when endophyte hyphae could be observed within the aleuron layer of the caryopsis. Four replicates of 25 seeds per ecotype were analysed. Results are expressed as a mean percentage of infected seeds (Se).

Seeds of selected, 18 endophyte infected (E+) ecotypes were sown into pots filled with sterilized peat substrate, placed in a greenhouse and watered as needed for 6 weeks. The experiment was conducted in 4 replicates (a single pot constitutes one replication). After 6 weeks of growth, twenty five seedlings per replication were sampled and microscopically examined for the presence of endophyte hyphae using the rose Bengal staining method described above [30]. Results are expressed as a mean percentage of infected plants (Ee) containing endophyte mycelium in each ecotype and refers to the intensity of colonisation.

### Horizontal transmission of *Neotyphodium lolii*

Formation of endophyte-free (E-) and endophyte-infected (E+) plants run in following steps:

**1-st step (both for E+ and E- plants) – cultivar selection and seed analysis**. Seed samples were kindly provided by seed producing companies in Poland. Four commercial cultivars of perennial ryegrass: Grilla, Maja, Nira and Vigor with confirmed different levels of infection by *N. lolii* were used in the presented research [15, 32]. According to immunoblot procedure [33] recommended by the International Seed Testing Association [34], seed accessions of two (low and high) contrasting levels of endophyte infection were selected: for Grilla −2% and 78%, for Maja −0% and 32%, for Nira – 0% and 45% and for Vigor – 0% and 90%. Samples of the lowest endophyte infection for each variety were further used to obtain endophyte-free (E-), as well as samples of the highest endophyte infection were used to obtain endophyte hosting (E+) plants.

**2-nd step (only for E- plants) – endophyte elimination from seed samples of identified low or zero hyphae presence**. Endophyte-free (E-) plants were obtained by treating seeds of above mentioned E- seed samples with tebuconazole [32, 35].

**3-nd step (only for E- plants) – endophyte detection in young seedlings (microscopic examination)**. Treated seeds were sown in greenhouse, in pots filled with sterilized peat substrate, and 4-weeks-old, young seedlings were examined for the presence of endophyte using rose Bengal staining solution as described above [30]. After next few weeks of growth, well-developed, at least 5-tillers-containing E- plants were further examined for endophyte presence before planting in the field.

**4- th step (only for E- plants) – endophyte detection in young seedlings (DNA analysis)**. To be quite sure that our E- plants are completely free from endophytes, the molecular analyses were performed in the Department of Molecular Phytopathology, University of Technology and Life Sciences in Bydgoszcz, Poland according to Dombrowski *et al*. [36]. Fungal DNA was extracted from the plant tissue of 3 tillers per plant using DNeasy Plant Mini Kit (Qiagen, Hilden, Germany). DNA was amplified by PCR with *Taq* PCR Core Kit (Qiagen, Hilden, Germany).

The following primers, amplifying a region of the intron of the *tub2* (tubulin 2) gene specific for *Neotyphodium* spp were used:

IS-RS-5’(5’GAGCCCCTGATTTCGTAC-3’),

IS-NS-3’ (5’TTGAAGTAGACACTCATACGCTC-3’).

The PCR reaction was run on a Uno II thermocycler (Biometra, Germany). Cycling conditions were as follows: an initial denaturation at 94°C for 1 min, followed by 18 cycles of 94°C for 25 s, 73°C for 25 s, and 72°C for 3 min, followed by 32 cycles of 94°C for 25 s, 58°C for 1 min, and 72°C for 2 min. Final elongation was at 73°C for 15 min. Obtained products of PCR reaction were analysed by electrophoresis in 1% agarose gel containing ethidium bromide (0.2 μg ml^−1^). Plants of confirmed absence of endophyte DNA (E- plants) were used to establish part of the field experiment.

**2-nd step (only for E+ plants)**. For E+ combinations, untreated seeds were sown in greenhouse, in pots filled with sterilized peat substrate, and the resulting seedlings were planted without additional tissue examination of endophyte presence.

**Fig 1:**
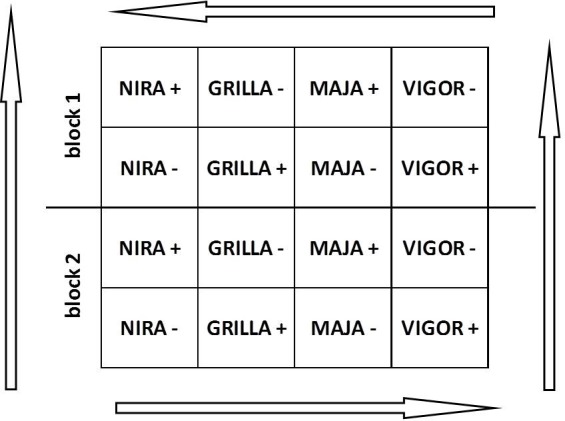
Layout of the field experiment on the horizontal transmission of endophytes (arrows indicate the mowing directions).

**Fig 2:**
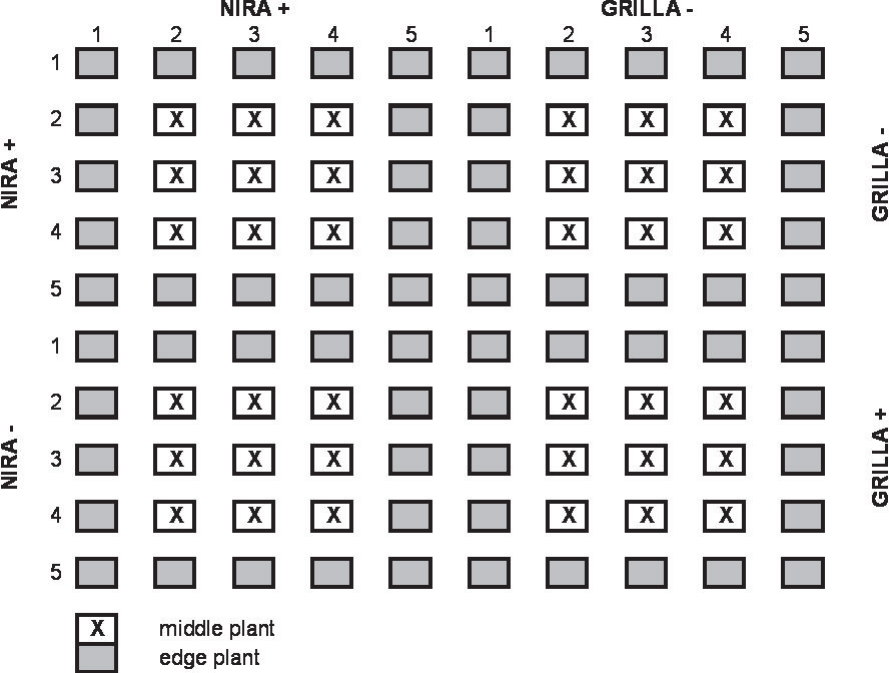
Example of a plot layout and the position of middle and edge plants.

**Field experiment**. Endophyte infected (E+) and endophyte free (E-) plants were planted in the field in plots that had 5 x 5 plants per plot with approx. 5 cm between plants within the plots and between the plots. Experiment with alternating plots of E+ and E- plants (Fig. 1) was set up on silt sandy soil (pH 6.7) in central Poland (Radzików, 52°12’ N, 20°37’ E) in a split-spilt-block design with two blocks. To support the possible spread of endophytes between plants, frequent mowing was applied. Typical rotary lawn mower (5.5hp, 4-stroke engine), with mowing width of ca. 43 cm were used 7 times per season. Mowing height was 5–7 cm, with clippings collected. Each time plots were mown in both directions, from left to right and from up to down. Above mentioned lawn mower was ascribed only to this experiment, and not used on another turf areas. After seven months of growth, five tillers per plant growing in the middle and at the edge of each plot (Fig. 2) were examined for the endophyte presence according to Dombrowski *et al*. [36], as described above.

### Statistical analyses

Statistical calculations were made according to SAS statistical package (SAS Institute Inc., Cary, NY, USA). The least significant differences (LSD) between means were calculated according to the Fisher honesty test and statistical significance was accepted at α = 0.05. The relationship between endophyte infection of seeds and that of the plants grown from these seeds was determined using Pearson’s correlation coefficient.

## Results and Discussion

### Vertical transmission of *Neotyphodium lolii*

Fourty six ecotypes of perennial ryegrass were collected: 16 in Podlaskie, 16 in Mazowieckie, and 14 in Świętokrzyskie. The highest endophyte presence in collected ecotypes was noted in populations from the Świętokrzyskie region (78.6%), whereas the lower in the Podlaskie and Mazowieckie regions (56.2% and 43.7%, respectively) (Table 1). Regional differences in the endophytic infections of perennial ryegrass might be due to different climatic conditions, which favour development of endophytes [37, 38]. Lewis [39] reported that in many cases in Europe, the incidence of endophytes in wild populations of *L. perenne* was high. Based on the results from many countries, he found that the highest incidence of endophytes was noted in plants of *F. arundinacea* (95%), whereas *F. pratensis* and *L. perenne* exhibited an approximately 40% lower (59% and 49%, respectively) infection frequency.

Further analysis demonstrated that the highest levels of *N. lolii* infection were noted in seed collected from ecotypes from the Świętokrzyskie and Mazowieckie regions. Mean Se values were 88.8% and 72.0%, respectively (Table 1). Relatively low level of colonisation (24.7%) was observed for seeds originating from the Podlaskie region. It was also found that the seeds of five ecotypes, four from Świętokrzyskie and one from Mazowieckie, were almost completely infected (Se >98%). On the other hand, the lowest values for seeds infection (Se<10%) were found in two ecotypes from the Podlaskie region and one ecotype from the Mazowieckie region.

Numerous studies have concluded that the degree of seeds infection by endophytes has a significant impact on the plant population in successive growing seasons [40, 41, 42], but this effect depends on many different factors [43, 44]. Wiewióra *et al*. [15] concluded that the age of a grass plantation that is grown for seed is important for the colonisation of seeds by fungi of the genus *Neotyphodium*. Research carried out by Hume and Barker [45] on pastures sown with *L. perenne* showed that even if these plants were established using E- seeds or seeds with a less severe infection, the severity of seed infection increased with the age of plantation. This phenomenon is attributed to residues of infected seeds in the soil or to selective cattle feeding of E- plants [45].

The mean endophyte infection of plants was lower in most of cases than that of seeds used in our experiment. Average differences between seed infection and infection of plants grown from those seeds ranged from 1.7% (Świętkorzyskie region) to 6.8% (Mazowieckie region). This finding is consistent with the view that the percentage of vital endophytes in plants is usually lower than the percentage found in seeds because of an incomplete transmission of endophytes into all tillers [17, 46].

**Fig 3:**
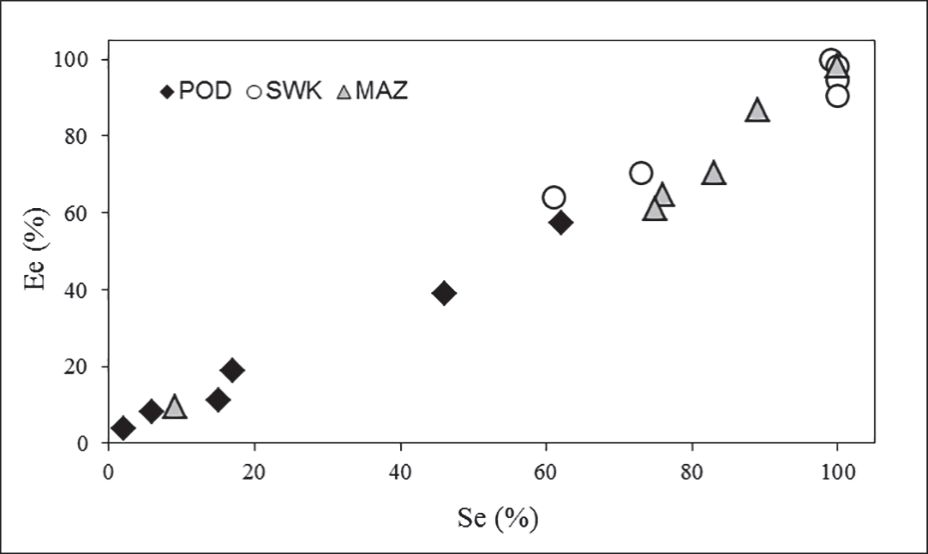
The relationship between endophyte infection rate of seeds (Se) and plants (Ee). Regions: POD – Podlaskie. SWK – Świętokrzyskie. MAZ – Mazowieckie.

A strongly positive correlation (*r* = 0.99) was observed between endophyte infection of seeds and that of the plants grown from these seeds (Fig. 3). It was therefore concluded that most endophytes in the seeds were vital and that the environmental conditions were suitable for vigorous growth of these fungi.

### Horizontal transmission of *Neotyphodium lolii*

The mean infection of E- plants growing at the edge of plots ranged from 60.0% to 90.0% (Table 2). Plants growing in the middle of the E- plots were also occupied by endophytes but to a lesser degree (from 5.0% to 20.0%). ‘Nira’ was the only cultivar for which the E- plants located in the middle of the plot were all free from endophytes after 7 months of growth. Statistically significant differences between the edge and middle plants were noted for all E- cultivars. The results of our experiment indicated that mean endophyte infection of tested plants was influenced by plant location on the field (edge or middle plants) as well as by endophyte status of plants at the onset of experiment (Table 3). It is therefore clear that the horizontal transmission of endophytes between plants occurred, especially between plants growing side-by-side.

**Table 2:** Mean endophyte infection of perennial ryegrass cultivars (%) after 7 months of mowing.

Cultivar	E+ plants			E- plants		
	EDG	MID	diff.	EDG	MID	diff.
GRILLA	77.5	95.0	ns	90.0	20.0	***
MAJA	77.5	40.0	**	72.5	20.0	***
NIRA	85.0	70.0	ns	70.0	0.0	***
VIGOR	100.0	90.0	ns	60.0	5.0	***
Mean	85.0	73.8	ns	73.1	11.3	***
LSD (α = 0.05)	15.3	14.1	-	10.8	10.5	-

Explanations: EDG – plants from the edge of plots, MID – plants from the middle of plots, diff. – significance of difference between EDG and MID plants: ns – not significant, significant with probability: **—P > 95%. ***—P>99%

**Table 3:** Results of ANOVA showing effects of cultivars, plant endophyte status, plant location and their interactions on mean endophyte infection of perennial ryegrass cultivars.

Source of variation:	df	MS effect	F-value	p
cultivar	3	519.5	5.6	0.008
endophyte status (E + or E-)	1	11063.3	119	0.000
plant location (EDG or MID)	1	10694.5	115	0.000
cultivar x endophyte status	3	875.8	9.4	0.001
cultivar x plant location	3	152.9	1.6	0.219
endophyte status x plant location	1	5125.8	55.1	0.000
cultivar x endophyte x location	3	446.6	4.8	0.014

Transmission of endophytes between plants might occur, and it is most likely related to the physical distance between E+ and E- plants. Transmission of endophyte hyphae might occur during frequent mowing or grazing (mower blade surface or animal hooves), through the root-to-root contact of plants or through herbivorous insect vectors [19]. Although the horizontal transmission of endophytes has not been previously demonstrated, the possibility of this phenomenon seems realistic because the inoculation of *Neotyphodium* into E- plants in the laboratory is a simple technique [47]. In two different species of grasses, White *et al*. [23] reported the natural occurrence of endophyte mycelium with conidiogenous cells and with conidia on the surfaces of leaves. This report suggests that without any external vectors affecting leaf structure, endophytic mycelia might exist outside of host tissues.

Fungal endophytes (ascomycete and coelomycete species) in oak trees were also reported to be horizontally transmitted by insect herbivores [48]. Iannone *et al*. [49] suggested that horizontal transmission of asexual endophytes from infected to non-infected hosts might have happened between grass plants or even sympatric grass species in South Africa.

The question concerning horizontal transmission of endophyte is the following: if this transmission occurs, then why are not all the perennial ryegrass populations infected in wild habitats? In our experiment, we grew plants in close proximity and mowed them to promote transmission of the endophyte. In nature, where plants can grow in patches or distant from one another, horizontal transmission by hyphae might not occur without frequent mowing, trampling, grazing or other such activities. Saikkonen *et al*. [50] stated that in heterogeneous environments with non-identical patches (e.g. wild grass populations highly structured in space), the coexistence of E+ and E- plants can occur, even if the endophyte is non-mutualistic or parasitic. Even the infrequent, sporadic horizontal transmission of an endophyte might be of critical importance for the survival and distribution of the fungus [50].

## Conclusions

Our results indicate that the vertical transfer of endophytes from seeds to plants of wild perennial ryegrass populations was almost complete. The degree of endophyte colonization of plants was directly related to the degree of colonization of seeds from which the plants grew. Moreover, the horizontal transmission of endophytes between plants of *Lolium perenne* was experimentally confirmed, which to our knowledge, has not been previously documented. Further research explaining different range of endophyte transmission depending on cultivar, initial level of seeds infection and plant location in the plot are needed.
